# Composition, Structure, and Techno-Functional Characteristics of the Flour, Protein Concentrate, and Protein Isolate from Purslane (*Portulaca oleracea* L.) Seeds

**DOI:** 10.1007/s11130-022-01028-4

**Published:** 2022-11-10

**Authors:** Ahmed M. Rayan, Hesham M. Swailam, Yahya S. Hamed

**Affiliations:** 1 Food Technology Department, Faculty of Agriculture, Suez Canal University, Ismailia, 41522 Egypt; 2 National Center for Radiation Research, and Technology (NCRRT), Atomic Energy Authority (AEA), Cairo, Egypt

**Keywords:** Composition, Purslane seeds, Protein concentrate, Protein isolate, Techno-functional characteristics

## Abstract

One potential avenue to increase the production of valuable protein ingredients for the food industry is developing vegetable proteins from non-traditional plant sources. In the present study, the composition, structure and techno-functional characteristics of defatted purslane flour (DF), protein concentrate (PC), and protein isolate (AP) were investigated. The results revealed that DF, PC, and PI contained low levels of moisture, ash, and fat. However, there were significant differences (*P* < 0.05) in protein content between DF, PC, and PI (32.9, 60.8 and 90.9%, respectively). The techno-functional characteristics of purslane proteins were enhanced by processing purslane flour into PC and PI products. Furthermore, the ratios of total essential amino acids to total amino acids in purslane protein samples were well above that stated for ideal food proteins. SDS-PAGE analysis indicated three subunits of protein in DF, PC, and PI. Scanning electron microscopy revealed that DF exhibited a compact-like structure; PC had a small, flaky, but porous type of particle, and PI had an intact flake-like structure. The FTIR analysis revealed that some alterations in the secondary structure of protein were occurred. In summary, purslane proteins can be considered new functional food ingredients with different nutritional and technological characteristics.

**Supplementary Information:**

The online version contains supplementary material available at 10.1007/s11130-022-01028-4.

## Introduction

Purslane (*Portulaca oleracea* L.) is a well-known important plant that belongs to the *Portulacaceae* family and naturally occurs as a weed in field crops and lawns [1]. It can be found in temperate and tropical regions worldwide. The aerial portion of purslane plant (leaves and stems) are the principal edible parts, which have been investigated for their chemical composition and nutrients in several studies [2, 3]. Purslane has recently been referred to as “functional food or power food” due to its high nutritive value [4]. Furthermore, its medicinal and therapeutic benefits have been well documented [5]. Its importance in herbal medicine stems from its use as a purgative, heart tonic, emollient, and muscle relaxant, as well as anti-inflammatory, and diuretic medications. Purslane also has been used to treat psoriasis and osteoporosis. Purslane is referred to as a "Global Panacea" and is one of the most popular medicinal plants, according to the World Health Organization (WHO). According to El-Sayed et al. [4] and Petropoulos et al. [6], purslane can be used as a raw material to make food products such as dairy products, and as an ingredient for many salad dishes. Compared to other plants, purslane has good nutritional value since it contains high levels of minerals, carbohydrates, proteins, *α*-tocopherol, ascorbic acid, omega-3 fatty acids, *ß*-carotene, glutathione and phenolic compounds.

Purslane seeds are eaten by humans and animals based on ethnobotanical studies, and they have similar medicinal characteristics to the aerial portion. Despite the high nutritional value of purslane in the human diet, its consumption as a green leafy vegetable is limited due to the accumulation of oxalic acid [6]. It has been reported that the aerial part of the plant contains higher amount of oxalic acid than the seeds [3]. Linoleic and linolenic acid levels in the seeds and seedcakes is balanced (33.80–34.74% and 32.83–34.64%, respectively) [6]. The nutritional value and antioxidant content of purslane make it a valuable food for humans. Therefore, the herb may be used in the future due to its exceptional nutritional potential [7]. Jalali et al. [8] and Nazeam et al. [9] investigated the antihyperlipidemic properties and antioxidant activities of purslane seeds. Phenolic compounds including protocatechuic and *p*-hydroxybenzoic acids and phenolic lipids such as alkylresorcinols are also found in the seeds [10]. The primary components of dried purslane seeds are fat (15.03%), carbohydrates (53.43%), and protein (27.58%) (w/w) [6].

Several technological solutions have been developed to create high-quality protein-rich foods using unusual vegetable source ingredients. Alternative protein sources, particularly from plants, recently have received considerable interest. In this regard, several studies have evaluated the qualities of novel proteins derived from unused seeds of a broad range of plants. Seeds of almond, kenaf, grape, watermelon and acacia have all been identified as new, cheap protein sources with good nutritional, technological, and physicochemical qualities [11–14]. However, there is a scarcity of research on the amino acid composition, physicochemical and functional characteristics of purslane seed proteins, which is critical for utilizing the seeds as a possible food additive or other food product. It has been reported that food processing, preparation, storage, and consumption behaviors are governed by the physicochemical characteristics of their proteins, known as their techno-functional characteristics [13]. Furthermore, the nutritional and quality characteristics of purslane seed proteins have not been compared to those of other commercial proteins. Therefore, the current study investigated the techno-functional characteristics, structure and amino acid composition (compared to FAO/WHO recommendations) of the DF, PC and PI from purslane seeds.

## Materials and Methods

The materials and methods are presented as supplementary material.

## Results and Discussion

### Chemical Composition

The chemical compositions of DF, PC and PI are shown in Table 1. The DF, PC and PI contained low levels of moisture (8.09, 5.18 and 4.84%, respectively), ash (5.84, 3. 91 and 3.58%, respectively) and fat (1.88, 0.65 and 0.56%, respectively). PI had the lowest values for all of these components. Accordingly, because of the low moisture content, purslane proteins are less sensitive to microbial attack. Purslane proteins are also acceptable for human consumption and animal feed due to their low ash levels, which are within the normal range for certain common edible seeds. The low concentrations of fat in purslane proteins could be attributed to their lipoprotein association, and these polar lipids play a key role in the flavor of the proteins [15].

**Table 1:** Chemical composition and techno-functional characteristics of defatted purslane flour (DF), protein concentrate (PC) and protein isolate (PI)^*^

Component	DF	PC	PI
Chemical composition			
Moisture (%)	8.09 ± 0.09^a^	5.18 ± 0.04^b^	4.84 ± 0.16^c^
Ash (%)	5.84 ± 0.16^a^	3.91 ± 0.05^b^	3.58 ± 0.08^c^
Fat (%)	1.88 ± 0.07^a^	0.65 ± 0.03^b^	0.56 ± 0.02^b^
Protein (%)	32.9 ± 0.82^c^	60.8 ± 0.37^b^	90.91 ± 0.45^a^
Carbohydrates (%)	59.38 ± 1.74^a^	34.66 ± 0.97^b^	4.95 ± 0.37^c^
Techno-functional characteristics			
Water absorption capacity (g/g)	5.28 ± 0.35a	4.16 ± 0.57b	2.94 ± 0.17c
Oil absorption capacity (g/g)	1.18 ± 0.03^b^	1.12 ± 0.022^b^	4.64 ± 0.27^a^
Foaming capacity (%)	3.91 ± 0.05^c^	12.27 ± 0.25^b^	23.96 ± 0.45^a^
Foaming stability (%)	11.66 ± 0.86^c^	26.83 ± 0.76^b^	46.05 ± 1.25^a^
Emulsifying activity (%)	11.50 ± 0.56^c^	20.83 ± 0.66^b^	52.33 ± 1.07^a^
Emulsifying stability (%)	15.33 ± 0.60^c^	33.11 ± 0.83^b^	57.66 ± 1.52^a^

*Results represent the average of three determinations ± SD, values in the same row with different letters are significantly different (*p* < 0.05)

Protein content differed significantly (*P* < 0.05) across DF, PC, and PI (32.9, 60.8, and 91.9%, respectively), with PI exhibiting the highest protein content. The high protein values of purslane seeds should encourage its use as a good source of protein in food formulations and could be considered an animal protein substitute to address the world's expanding protein need. Furthermore, the protein recovery yields of extracted concentrates and isolates were 55 and 15% of the flour, respectively. The higher recovery yield of PC than PI might be attributed to the non-proteinaceous material remaining during the concentrate preparation. DF, PC, and PI had significantly different carbohydrate contents (*P* < 0.05), with values of 59.38, 34.66, and 4.95%, respectively. The variations in chemical composition between the DF, PC, and PI could be attributable to the protein extraction procedures. As a result, the extraction procedures boosted the protein levels while decreasing the other components. The current findings are consistent with those obtained for groundnut proteins [16], walnut PC and PI [17] and acacia PC and PI [14]. These observations might increase interest in using purslane as a high protein source in some food formulations.

### Techno-Functional Characteristics

#### Protein Solubility

**Fig. 1:**
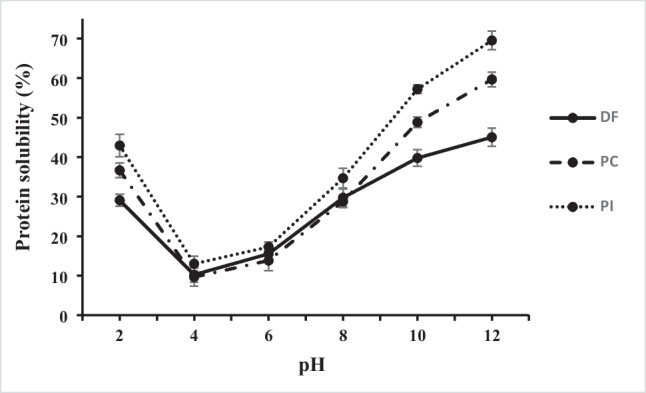
Protein solubility profile of defatted flour (DF), protein concentrate (PC), and protein isolate (PI) of purslane seeds

The protein solubility profile of DF, PC and PI at several pH levels (2 – 12) is illustrated in Fig. 1. At pH 4, DF had a minimum solubility of 10.23%, while the solubility was 29.1% at pH 2. From pH 6 to pH 12, the solubility of DF increased progressively, with the greatest solubility (45.0%) at pH 12. Similarly, the solubility at different pH ranges for PC was investigated. Where at pH 2, the solubility of PC was 36.6%, and then dropped to the lowest level (9.64%) at pH 4. Subsequently, the solubility was raised from pH 6 to 12, reaching a high level of 59.6% at pH 12. Like DF and PC, PI showed high solubility at the acidic and alkaline pHs, with the lowest solubility of 13.0% at pH 4 and the highest solubility of 69.5% at pH 12. The high solubility of PI can be attributed to the extraction occurring at an alkaline pH, which presents a group of proteins that have already been subjected to alkaline unfolding. In addition, they were relatively free from most of interfering components. Finally, purslane proteins have low solubility at pH 4, indicating that they are acidic in nature and that the protein's isoelectric point is at pH 4. This was confirmed by the maximum protein content of purslane protein produced using the isoelectric precipitation method. In fact, because the majority of dietary proteins are acidic in nature, their solubility is lowest around pH 4–5, increasing as the pH value rises [18]. Furthermore, the protein is less soluble at the isoelectric point (pI) due to peptides' net charge and the surface hydrophobicity [19]. Protein solubility is complex and could be affected by different factors, such as electrostatic/hydrophobic interactions and hydrogen bonding. These parameters could promote protein–protein interactions or protein-solvent interactions [20].

#### WAC

As illustrated in Table 1, the WACs of DF, PC, and PI were significantly different (*P* < 0.05). DF showed the highest WAC of 5.28 (g/g) followed by PC (4.16 g/g) compared with PI (2.94 g/g). This could be due to the greater carbohydrate levels of DF and PC, which contribute to the WAC. The same observations have been reported by Pedroche et al. [21] for defatted meals of seeds of *B. carinata*, Vioque et al. [22] for *V. faba* and Bernardino-Nicanor et al. [23] for *Pouteria sapota* seed meal. The WAC values of PC and PI were high compared to those obtained by Bernardino-Nicanor et al. [23] for *Pouteria sapota* seed meal, Pedroche et al. [21] for *B. carinata* seeds, Zhu et al. [24] for commercial soy PI and Jain et al. [16] for groundnut PC and PI. On the other hand, the WAC value obtained for DF is similar to that obtained by Al-Kahtani and Abou Arab [20] for moringa and Carvalho et al. [25] for cupuassu protein concentrate and isolate. The variations in WACs for the investigated samples might be due to the nature and type of protein as well as the hydrophilic properties of proteins that are related to polar groups located on the primary sites of protein-water interactions, including carbonyl, hydroxyl, amino, sulfhydryl and hydroxyl groups [17]. Lawal et al. [26] reported that intrinsic factors influenced the WHC of food proteins including amino acid composition, protein conformation, surface polarity and hydrophobicity. The high WAC of purslane PC and PI makes them a useful ingredient in food applications. The ability of proteins to bind water and oil is a crucial technological feature that allows them to physico-chemically integrate with the other macro and micro components of food, improving qualities like texture, mouthfeel, and flavour retention [25].

#### OAC

The OACs of DF, PC, and PI were 1.18, 1.12 and 4.64 g oil/g, respectively (Table 1). The OACs for DF, PC and PI were lower than that of cupuassu [25] for DF (3.46 g/g), PC (5.77 g/g), and PI (17.2 g/g) but similar to that reported for moringa, walnut and groundnut flours, protein concentrate and isolate (the values ranged from 1.36 – 3.48 ml/g) [16, 17, 20]. Using an alkaline pH during protein isolation might be responsible for the high OAC of PI. Protein denaturation could result from the alkaline environment. Because of the denaturation process, globular proteins can be opened, allowing hydrophobic amino acids like leucine, isoleucine, phenylalanine, methionine, tryptophan, and valine to bind fat easily. Generally, proteins have the potential to bind fat, allowing them to enhance the flavor and texture retention of food products [27]. The mechanism of protein binding to fats could be attributed partially to physical entrapment and is associated with bulk density and particle size. Furthermore, the high OAC of purslane PI makes it an excellent ingredient in cold meat manufacturing since the protein has the ability to enclose fat and water in such foods [28].

#### Foaming Characteristics

Foaming capacity (FC) and foaming stability (FS) for DF, PC and PI are illustrated in Table 1. According to the results, PI had a higher level of FC (23.96%) than PC (12.27%) and DF (3.91%). The lower FC of DF and PC was likely due to their more complicated matrix than PI. Like FC, the FS of PI (46.05%) was significantly higher (*P* < 0.05) than PC (26.83%) and DF (11.66%). The obtained results were superior to those previously reported for grape seeds protein [12]. However, the results were similar to those reported by Ogunwolu et al. [28] for cashew nut powder and Mao and Hua [17] for walnut flour. Several parameters, such as molecular transit, penetration, and restructuring at the air/water interface, govern foam formation [28]. To promote effective foaming, proteins should be capable of migrating at the contact between air and water, unfolding and reforming at the interface [12]. Overall, the high FC and FS of PI help to use it in food systems that require foaming such as cakes and ice cream.

#### Emulsifying Properties

The PI had the highest emulsifying activity (EA) level (52.33%), followed by PC (20.83%) and that of DF (11.50%) was the lowest. Similarly, ES of PI was the highest (57.66%), followed by PC (33.11%) and DF (15.33%) was the lowest with significant differences (*P* < 0.05) (Table 1). Carvalho et al. [25] discovered similar results for cupuassu PC and PI. This result might have been caused by the isolated molecules adapting to the oil–water interphase more effectively than the native proteins in the flour and concentrate that were still linked to the non-proteinaceous material. It also might have occurred because the isolated molecules stripped of their associated non-proteinaceous material by the alkaline unfolding. When exposed to only water or oil or a combination of water and oil, it appears that each product responded in a manner that was unique [25]. The ability of a protein to assist the dispersion of an oil phase into an aqueous medium is measured by its emulsifying capability [12]. The high emulsifying activity of purslane seed protein isolate might make it suitable for food applications especially foods depend on emulsification, including meat products and mayonnaise.

Data in Table S1, explain the correlation coefficient among the techno-functional characteristics of purslane proteins. There were significant and negative correlations between the WAC with OAC, FA, FS, EA and ES. On the other hand, there were highly significant and positive correlations between all other techno-functional characteristics with each other.

### Amino Acid Composition

The amino acid content of proteins is a crucial chemical feature since it dictates the nutritive value of proteins. The amino acid compositions of DF, PC and PI of purslane seeds are illustrated in Table 2. When the amino acid composition of purslane seed proteins was compared to the FAO/WHO [29] recommended content, it was evident that the essential amino acids content was higher than the adult-recommended levels. In addition, DF, PC, and PI contained high levels of valine, leucine, tyrosine, phenylalanine and threonine. The essential amino acids content was comparable to the FAO/WHO [29] recommended content for pre-school children; except methionine, tyrosine/phenylalanine and histidine in the DF, PC and PI of purslane seeds were low. The amounts of all essential amino acids in PI, on the other hand, were higher than in DF and PC. The differences in amino acid contents might be attributed to the chemical alterations in some amino acids due to the extraction process, which included by alkaline and acids treatments [30]. Glutamic acid, aspartic acid, and arginine were the predominant non-essential amino acids in all protein samples. The amino acid arginine is essential for children. The values of arginine (6.883, 5.734 and 6.756 g/100 g protein for DF, PC and PI, respectively) were similar to the reference protein (6.10 g/100 g) [31].

**Table 2:** Amino acid composition of defatted purslane flour (DF), protein concentrate (PC), and protein isolate (PI)

Amino acid^*^	DF	PC	PI	FAO/WHO reference^a^
Essential				
Methionine	2.242	2.011	2.725	2.5 (1.7)
Cystine	ND	ND	ND	
Valine	2.183	2.454	2.246	3.5 (1.3)
Isoleucine	1.990	1.212	2.079	2.8 (1.3)
Leucine	4.667	3.606	5.559	6.6 (1.9)
Tyrosine	3.232	3.895	3.789	6.3 (1.9)
Phenylalanine	5.469	3.357	5.334	
Tryptophan	ND	ND	ND	
Histidine	1.641	2.101	2.092	1.9 (1.6)
Lysine	0.892	1.694	1.551	5.8 (1.6)
Therionine	2.545	2.445	2.722	3.4 (0.9)
Nonessential				
Aspartic acid	6.311	4.768	7.109	
Proline	0.703	0.357	0.438	
Serine	3.262	2.824	3.649	
Glutamic acid	9.950	10.01	14.42	
Glycine	4.332	3.948	3.961	
Alanine	2.896	2.666	4.429	
Arginine	6.883	5.734	6.756	

^*^All amino acid (AA) values are expressed as grams *per* 100 g of protein. ^a^ Numbers in parentheses of FAO/WHO recommended content [29] represent essential amino acid for adults and numbers outside the parentheses represent essential amino acid for pre-school children (2~5 years). ND; not detected

The ratios of total essential amino acids (TEAA) to total amino acids (TAA) in purslane protein samples were 41.9, 42.9 and 40.8% for DF, PC and PI, respectively, which above the level stated for ideal protein-containing food for infants (39%), children (26%) and adults (11%) [29, 32].

### SDS-PAGE

The protein structure's components (subunits) are primarily responsible for its functionality [16]. SDS-PAGE patterns of purslane proteins are illustrated in Fig. (S1) (molecular weight standards were 10, 15, 25, 35, 55, 70, 100, 130, and 250 kDa). The electrophoretic pattern of DF, PC and PI was comparable, indicating that the majority of the polypeptides found in DF also were found in both PC and PI with molecular weights of ~ 12, 17 and 30 kDa.

These obtained results are in accordance with those reported for walnut proteins by Mao and Hua [17] who found that the SDS-PAGE profiles of walnut DF, PC and PI exhibited no notable qualitative variations, indicating that the alkaline extraction-isoelectric precipitation approach and the isoelectric precipitation technique did not modify the protein composition. It was suggested that the electrophoretic velocities depend on different factors, including the pH of the separating buffer, as well as the retardation coefficient associated with the shape and size, charge, and structure of the neighboring charged particles [16, 33]. Al-Kahtani and Abou-Arab [20] stated that the changes in electrophoretic pattern might be attributed to the processing of DF into PC and PI.

### Microstructure

Scanning electron micrographs of DF, PC and PI are shown in Fig. (S2). DF revealed a dense cell structure comprising a mixture of fat particles and starch granules surrounded by a protein matrix (Fig. S2 A). PC had a small, flaky, but porous type of particles (Fig. S2 B). PI had an intact flake-like structure because it was made up entirely of protein (Fig. S2 C). The obtained microstructures are comparable to those observed for DF, PC and PI from acacia [14], groundnut [16], and walnut [17], respectively. The porous and flake-like structures of both PC and PI have been shown to promote protein digestion and solubility [16, 17]. The various microstructures of DF, PC, and PI might be influenced by physiochemical and techno-functional characteristics.

### FTIR Analysis

The FTIR analysis revealed that the obtained peak positions of purslane proteins had a place for amide-Ι (1640, 1641, and 1636 cm^−1^), amide-ΙΙ (1528, 1533, and 1528 cm^−1^) and amide-ΙΙΙ (1386, 1390, and 1389 cm^−1^) for DF, PC, and PI, respectively, due to the presence of C = O, N–H, C–H, and N–H groups (Fig. S3 and Table S2). The region of the mid-infrared spectrum (1300 to 1700 cm^−1^) has been determined to be suitable for studying proteinaceous materials where the absorption of the amide groups is within this range [34, 35]. The strongest absorption band for polypeptides among the several protein amide bands is the amide-I from 1600 to 1700 cm^−1^ [35]. The differences in band intensity of DF, PC, and PI, indicate that the quantity of protein might have had an impact on the active groups of the amino acids. Accordingly, some alterations in the secondary protein structure (*α*-helix, *β*-turn) might be occurred. It could be concluded that the processing of purslane seed flour into PC and PI increased the intensity of amide groups, might have changed the protein’s secondary structure and improved the functional and technological characteristics of protein matrices.

## Conclusion

The results indicated that the chemical composition and structure of PC and PI of purslane were different compared to DF. Furthermore, the techno-functional characteristics of purslane flour were enhanced by processing into PC and PI. The ratios of total essential amino acids to total amino acids in purslane protein samples were well above that determined in ideal protein for infants, children and adults. Furthermore, the microstructures, including porous particles and flakes, for both PC and PI would promote protein digestion and solubility. The improved techno-functional characteristics of processed purslane PC and PI are expected to influence the quality characteristics of food products and could be used as a potential source of nutrients supplementation in some functional food products. We recommend additional investigation into the potential use of purslane proteins in suitable food products, depending on their techno-functional properties.

## Supplementary Information

Below is the link to the electronic supplementary material.

Supplementary file1 (DOCX 101 KB)

Supplementary file2 (DOCX 1115 KB)

Supplementary file3 (DOCX 26 KB)

## Data Availability

Data is available upon request.

## Abbreviations

AOAC: Association of Official Analytical Chemists
DF: Defatted purslane flour
DIW: De-ionized water
DW: Distilled water
EA: Emulsifying activity
ES: Emulsifying stability
FC: Foaming capacity
FS: Foaming stability
FTIR: Fourier Transform Infrared spectroscopy
OAC: Oil absorption capacity
PC: Protein concentrate
PI: Protein isolate
SDS-PAGE: Sodium dodecyl sulfate–polyacrylamide gel electrophoresis
WAC: Water absorption capacity

## References

[1] Petropoulos S, Karkanis A, Martins N, Ferreira ICFR (2016). Phytochemical composition and bioactive compounds of common purslane (*Portulaca oleracea* L.) as affected by crop management practices. Trends Food Sci Technol.

[2] Petropoulos S, Karkanis A, Fernandes Â, Barros L, Ferreira ICFR, Ntatsi G (2015) Chemical composition and yield of six genotypes of common purslane (*Portulaca oleracea* L.): an alternative source of omega-3 fatty acids. Plant Foods Hum Nutr 70:420–426. 10.1007/s11130-015-0511-8 10.1007/s11130-015-0511-8 26510561

[3] Petropoulos SA, Fernandes A, Dias MI, Vasilakoglou IP, Petrotos K, Barros L, Ferreira ICFR (2019). Nutritional value, chemical composition and cytotoxic properties of common purslane (*Portulaca oleracea* L.) in relation to harvesting stage and plant part. Antioxidants.

[4] El-Sayed MI, Ibrahim AA, Awad S (2019) Impact of purslane (*Portulaca oleracea* L.) extract as antioxidant and antimicrobial agent on overall quality and shelf life of Greek-style yoghurt. Egypt J Food Sci 47: 51- 64. 10.21608/EJFS.2019.12089.1005

[5] Gonnella M, National I, Charfeddine M, Agricultural CRA, Universit GC, Universit PS (2010) Purslane: a review of its potential for health and agricultural aspects. Eur J Plant Sci Biotechnol 4:131–136

[6] Petropoulosa SA, Fernandesb A, Arampatzisa DA, Tsiropoulosa NG, Petrovićc J, Sokovićc M, Barrosb L, Ferreirab ICFR (2020) Seed oil and seed oil byproducts of common purslane (*Portulaca oleracea* L): a new insight to plant-based sources rich in omega-3 fatty acids. LWT-Food Sci Technol 123:109099. 10.1016/j.lwt.2020.109099

[7] Uddin MK, Juraimi AS, Hossain MS, Nahar MA, Ali ME, Rahman MM (2014). Purslane weed (*Portulaca oleracea*): a prospective plant source of nutrition, omega-3 fatty acid, and antioxidant attributes. Sci World J.

[8] Jalali MSR, Niazmand R, Shahidi NM (2015). Antioxidant activity of purslane (*Portulaca oleracea* L.) seed hydro-alcoholic extract on the stability of soybean oil. J Agric Sci Technol A.

[9] Nazeam JA, El-Hefnawy HM, Omran G, Singab AN (2018). Chemical profile and antihyperlipidemic effect of *Portulaca oleracea* L. seeds in streptozotocin-induced diabetic rats. Nat Prod Res.

[10] Gunenc A, Rowland O, Xu H, Marangoni A, Hosseinian F (2019). *Portulaca oleracea* seeds as a novel source of alkylresorcinols and its phenolic profiles during germination. LWT-Food Sci Technol.

[11] Mariod AA, Fathy SF, Ismail M (2010). Preparation and characterization of protein concentrates from defatted kenaf seed. Food Chem.

[12] Zhou T, Zhang T, Liu W, Zhao G (2011). Physicochemical characteristics and functional properties of grape (*Vitis vinifera* L.) seeds protein. Int J Food Sci Technol.

[13] Wani AA, Sogi DS, Singh P, Wani IA, Shivhare US (2011). Characterization and functional properties of watermelon (*Citrullus lanatus*) seed proteins. J Sci Food Agric.

[14] Embaby HE, Swailam HM, Rayan AM (2018). Preparation and physicochemical properties of protein concentrate and isolate produced from *Acacia tortilis* (Forssk.) Hayne ssp. raddiana. J Food Sci Technol.

[15] Sanchez-Vioque R, Clemente A, Vioque J, Bautista J, Millan J (1999). Protein isolation from chickpea (*Cicer arietinum* L): chemical composition, functional properties and protein characterization. Food Chem.

[16] Jain A, Prakash M, Radha C (2015). Extraction and evaluation of functional properties of groundnut protein concentrate. J Food Sci Technol.

[17] Mao X, Hua Y (2012). Composition, structure and functional properties of protein concentrates and isolates produced from Walnut (*Juglans regia L*.). Int J Mol Sci.

[18] Damodaran S, Damodaran S, Paraf A (1997). Food proteins: an overview. Food proteins and their applications.

[19] Lautenbach V, Hosseinpour S, Peukert W (2021). Isoelectric point of proteins at hydrophobic interfaces. Front Chem.

[20] Al-Kahtani HA, Abou-Arab AA (1993). Comparison of physical, chemical, and functional properties of *Moringa peregrina* (Al-Yassar or Al-Ban) and soybean proteins. Cereal Chem.

[21] Pedroche J, Yust MM, Lqari H, Giron-Calle J, Alaiz M, Vioque J, Millan F (2004) *Brassica carinata* protein isolates: chemical composition, protein characterization and improvement of functional properties by protein hydrolysis. Food Chem 88:337–346. 10.1016/j.foodchem.2004.01.045

[22] Vioque J, Alaiz M, Girón-Calle J (2012). Nutritional and functional properties of *Vicia faba* protein isolates and related fractions. Food Chem.

[23] Bernardino-Nicanor A, Bravo-Delgado CH, Vivar-Vera G, Martínez-Sánchez CE, Pérez-Silva A, Rodríguez-Miranda J, Vivar-Vera MA (2014). Preparation, composition, and functional properties of a protein isolate from a defatted mamey sapote (*Pouteria sapota*) seed meal. CyTA-J Food.

[24] Zhu KX, Sun XH, Chen ZC, Peng W, Qian HF, Zhou HM (2010). Comparison of functional properties and secondary structure of defatted wheat germ proteins separated by reverse micelles and alkaline extraction and isoelectric precipitation. Food Chem.

[25] Carvalho AV, Garcia NHP, Amaya-Farfan J (2006) Physico-chemical properties of the flour, protein concentrate, and protein isolate of the cupuassu (*Theobroma grandiflorum* Schum) seed. J Food Sci 71(8):S573. 10.1111/j.1750-3841.2006.00156.x

[26] Lawal OS, Adebowale KW, Ongunsanwo BM, Sosanwo OA, Bankole SA (2005). On the functional properties of globulin and albumin protein fractions and flour of African locust bean (*Parkia biglobossa*). Food Chem.

[27] Kinsella JE, Damodaran S, German B (1985). Physicochemical and functional properties of oil seeds proteins with emphasis on soy proteins. New Protein Foods.

[28] Ogunwolu SO, Henshaw FO, Mock HP, Santros A, Awonorin SO (2009). Functional properties of protein concentrates and isolates produced from cashew (*Anacardium occidentale* L.) nut. Food Chem.

[29] FAO/WHO (1990) Protein quality evaluation. In: Report of a joint FAO/WHO expert consultation. Food and Agriculture Organization of the United Nations, Rome, p 23

[30] Moure A, Sineiro J, Dominguez H, Parajo JC (2006). Functionality of oilseed protein products: a review. Food Res Int.

[31] Fasuan TO, Gbadamosi SO, Omobuwajo TO (2018) Characterization of protein isolate from *Sesamum indicum* seed: *in vitro* protein digestibility, amino acid profile, and some functional properties. Food Sci Nutr 6:1715–1723. 10.1002/fsn3.743 10.1002/fsn3.743 PMC6145213 30258616

[32] Olaofe O, Adeyeye EI, Ojugbo S (2013). Comparative study of proximate, amino acids and fatty acids of *Moringa oleifera* tree. Elixir Appl Chem.

[33] Wolf W (1977) Legumes: seed composition and structure, processing into protein products and protein properties. Food Proteins / J. R. Whitaker and S. R. Tannenbaum, 291–314.

[34] Barakat H, Shams A, Denev P, Khalifa I (2022). Incorporation of quinoa seeds accession in instant noodles improves their textural and quality characteristics. J Food Sci Technol.

[35] Khalifa I, Nie R, Ge Z, Li K, Li C (2018). Understanding the shielding effects of whey protein on mulberry anthocyanin: Insights from multispectral and molecular modelling investigation. Int J Biol Macromol.

