# Supplementary material for: Composition, Structure, and Techno-Functional Characteristics of the Flour, Protein Concentrate, and Protein Isolate from Purslane (Portulaca oleracea L.) Seeds
Source: Plant Foods Hum Nutr. 2022 Nov 10;78(1):117–23. doi: 10.1007/s11130-022-01028-4 (PMC9947059; doi:10.1007/s11130-022-01028-4)
Supplement: Supplementary file 1 — Supplementary file1 (DOCX 101 KB) [file 11130_2022_1028_MOESM1_ESM.docx]

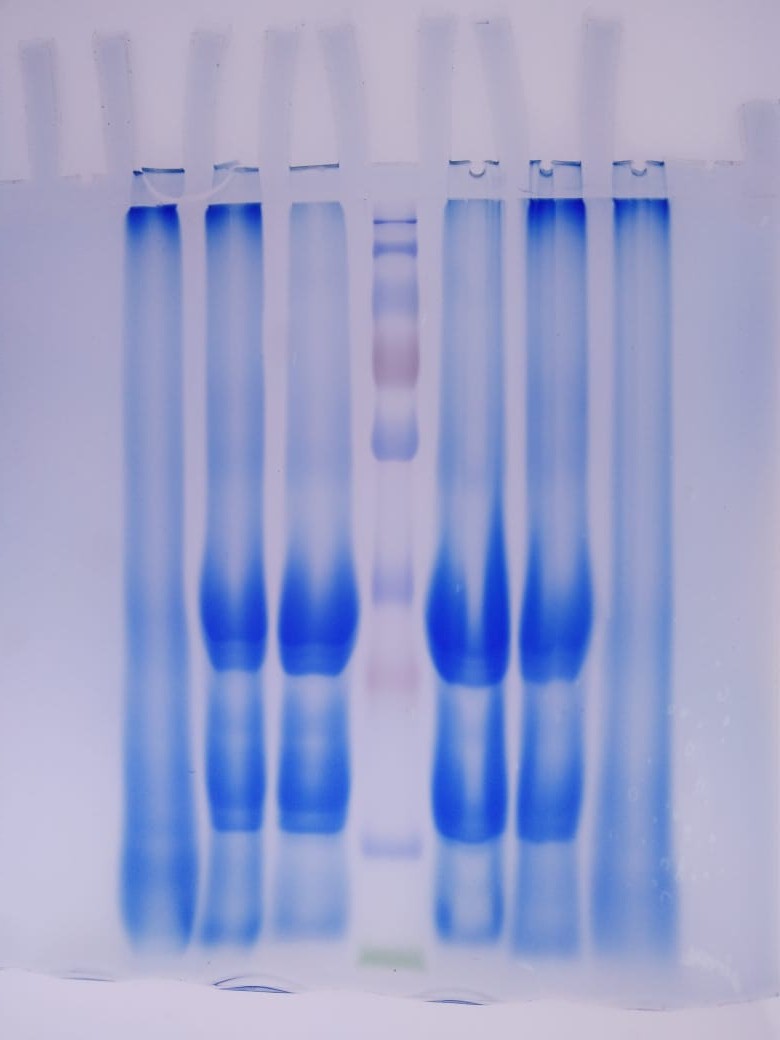


kDa

250

130

100

70

55

35

25

15

10

| PI | PC | DF | M | PI 1 | PI 2 | PI 3 |
| --- | --- | --- | --- | --- | --- | --- |

Replications for Protein isolate

**Fig. S1 SDS-PAGE pattern of purslane protein samples**. M: Molecular weight standards (10, 15, 25, 35, 55, 70, 100, 130 and 250 kDa); DF: defatted purslane flour; PC: purslane protein concentrate; PI: purslane protein isolate.
