# Supplementary material for: Composition, Structure, and Techno-Functional Characteristics of the Flour, Protein Concentrate, and Protein Isolate from Purslane (Portulaca oleracea L.) Seeds
Source: Plant Foods Hum Nutr. 2022 Nov 10;78(1):117–23. doi: 10.1007/s11130-022-01028-4 (PMC9947059; doi:10.1007/s11130-022-01028-4)
Supplement: Supplementary file 2 — Supplementary file2 (DOCX 1115 KB) [file 11130_2022_1028_MOESM2_ESM.docx]

| **A** | 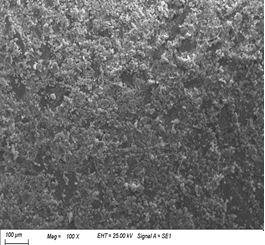 | 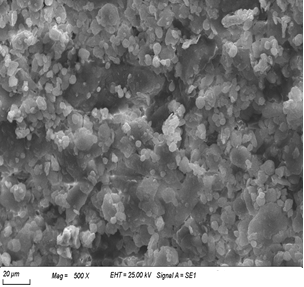 |
| --- | --- | --- |
| **B** | 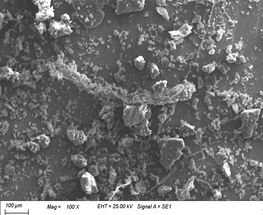 | 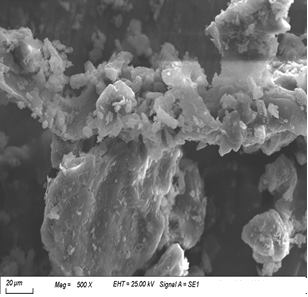 |
| **C** | 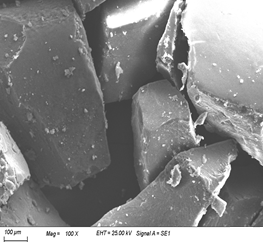 | 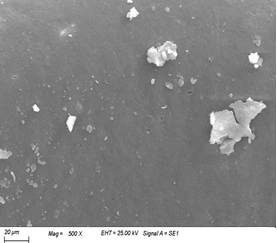 |

**Fig. S2** Scanning electron microscope pictures (magnification levels 100× and 500×) of defatted purslane flour (A), protein concentrate (B), and protein isolate (C)

**
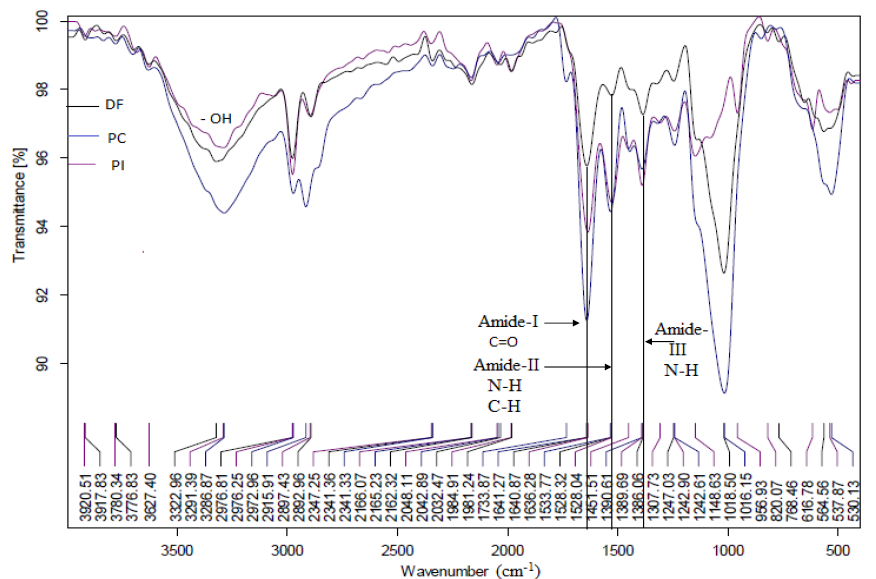
**

**Fig. S3** FTIR spectra of defatted purslane flour (DF), protein concentrate (PC), and protein isolate (PI)
