# Supplementary material for: Composition, Structure, and Techno-Functional Characteristics of the Flour, Protein Concentrate, and Protein Isolate from Purslane (Portulaca oleracea L.) Seeds
Source: Plant Foods Hum Nutr. 2022 Nov 10;78(1):117–23. doi: 10.1007/s11130-022-01028-4 (PMC9947059; doi:10.1007/s11130-022-01028-4)
Supplement: Supplementary file 3 — Supplementary file3 (DOCX 26 KB) [file 11130_2022_1028_MOESM3_ESM.docx]

**Materials and Methods**

**Materials**

Dry seeds of purslane (*Portulaca oleracea* L.) were purchased from the local market, Ismailia Governorate, Egypt. The whole purslane seeds were milled and the resultant flour was defatted using hexane (1 flour/ 10 hexane; w/v). The obtained DF was dried (in air) and ground to get size of 150 mesh and stored at −20°C until use.

**Methods**

**Preparation of protein concentrate**

Purslane seed PC was produced using the method of Wolf [1]. At ambient temperature, DF was blended with aqueous alcohol (95%; 1:20, w/v) then stirred for 1 h. After filtration of the suspension, the precipitate was air dried then mixed with de-ionized water (DIW) (1:20, w/v). The mixture was acidified to pH of 4.5 and stirred for 2 h using a magnetic stirrer. After centrifuging the slurry, the pellets were washed with DIW and neutralized to pH 7.0, then freeze-dried using a freeze dryer (Operon freeze-dryer, Operon Co. Ltd., Korea).

**Preparation of protein isolate**

The PI was produced using the method of Wolf [1]. The DF was blended with de-ionized water (1:20, w/v) then stirred for 2 h at ambient temperature and the pH was raised to 11.0 using NaOH (1 N). The mixture was centrifuged, and the pellets were re-dissolved in DIW and raised again to pH 11.0 then re-centrifuged. The obtained supernatants were combined and the pH was adjusted to 4.5 using HCl (1 N), then stirred for 2 h at ambient temperature and thereafter centrifuged. The pellets were washed and re-dissolved in water then the pH was adjusted to 7.0, the resultant slurry was freeze-dried.

**Proximate composition analysis**

Moisture, ash, fat, protein (N × 6.25) contents, and yields were carried out according to AOAC methods [2] and carbohydrates were calculated by difference. The data were shown as mean ± SD of three determinations.

**Physicochemical and techno-functional characteristics**

Solubility of purslane seed proteins were conducted in a pH ranged from 2.0 to 12.0 according to Rodrigues-Ambriz et al. [3]. Water absorption capacity (WAC) was performed using the method of Rodriguez-Ambriz et al. [3]. Oil absorption capacity (OAC) was performed using the method of Monteiro and Prakash [4]. Foaming capacity (FC) and foaming stability (FS) were determined by the procedure of Sze-Tao and Sathe [5]. Emulsifying activity (EA) and emulsion stability (ES) were measured using the method of Pedroche et al. [6].

**Amino acid analysis**

Protein samples were hydrolyzed using HCl-Phenol solution. The hydrolyzed mixture was evaporated and dissolved in citrate buffer (10 mL; 0.1 M, pH 2.2) then filtered and injected into the HPLC according to Jajić et al. [7]. The HPLC analyses were performed using an Agilent 1260 series. Eclipse Plus C18 column (3.0 mm x 150 mm i.d., 3.5 μm) was used for separation. The recovered amino acid ratios were calculated as (g /100 g) of protein and compared to the reference pattern [8].

**Polyacrylamide gel electrophoresis (PAGE)**

The SDS-PAGE was performed using the method of Laemmli [9]. Pursalne protein samples were dissolved in SDS–PAGE sample buffer in the presence of beta-mercaptoethanol. Samples were heated at 65°C for 30 mins and then subjected to centrifugation at 13,000 ×g to remove insoluble components. The concentrations were adjusted to ensure that each lane loaded with 30 µg of protein. The SDS–PAGE analysis was performed in duplicates.

**Scanning electron microscopy (SEM) analysis**

The DF, PC, and PI samples were spurted with gold after being placed on the silicon to a thickness of ~ 100 nm. The shape and surface characteristics of protein samples were inspected and recorded using SEM (ZEISS Evo 15, UK).

**FTIR analysis**

The FTIR spectra of purslane proteins were carried out by FTIR spectrometer (Bruker Optik GmbH, Ettlingen, Germany). The established lozenges were prepared after mixing the samples with KBr (1:99). The FTIR spectra were done at 21 scans per min with a transmission position of 4000 to 400 cm^-1^ and a clarity point of 2 cm^-1^.

**Statistical analyses**

All measurements were performed in triplicate and the obtained results are expressed as mean ± SD. The data were analyzed by ANOVA with Duncan test using SPSS software (version 16.0 for Windows, SPSS Inc., Chicago), with a significance level of p<0.05. Pearson’s correlation coefficient between the techno-functional characteristics was obtained through the software SPSS 16.0.

**References**

**1**. Wolf W (1977) Legumes: seed composition and structure, processing into protein products and protein properties. Food Proteins / J. R. Whitaker and S. R. Tannenbaum, 291-314.

**2**. AOAC (2005) Official methods of analysis, 18^th^ ed. Association of Official Analytical Chemists, Gaithersburg, MD.

**3**. Rodrigues-Ambriz S, Martinez-Ayala A, Millan F, Davila-Ortiz Z (2005) Composition and functional properties of *Lupinus campestris* protein isolates. *Plant Foods Hum Nutr* 60: 99-107. https://doi.org/10.1007/s11130-005-6835-z

**4**. Monteiro PV, Prakash, V (1994) Functional properties of homogeneous protein fractions from peanut (Arachis hypogaea L.). *J Agric Food Chem* 42: 274–278. <http://doi.org/10.1021/jf00038a009>

**5**. Sze-Tao KWC, Sathe SK (2000) Functional properties and in vitro digestibility of almond (*Prunus dulcis* L.) protein isolate. *Food Chem* 69: 153–160. <https://doi.org/10.1016/S0308-8146(99)00244-7>

**6**. Pedroche J, Yust MM, Lqari H, Giron-Calle J, Alaiz M, Vioque J, Millan F (2004) *Brassica carinata* protein isolates: Chemical composition, protein characterization and improvement of functional properties by protein hydrolysis. *Food Chem* 88: 337–346. <https://doi.org/10.1016/j.foodchem.2004.01.045>

**7**. Jajić I, Krstović S, Glamočić D, Jakšić S, Abramović B (2013) Validation of an HPLC method for the determination of amino acids in feed. J Serbian Chem Soc 78: 839–850. https://doi. org/10.2298/JSC120712144J

**8**. FAO/WHO (1990) Protein quality evaluation. In: Report of a joint FAO/WHO expert consultation. Food and Agriculture Organization of the United Nations, Rome, p 23

**9**. Laemmli UK (1970) Cleavage of structural proteins during the assembly of the head of bacteriophage T4. *Nature* 227: 680–685.

**Table S1 Correlation coefficient among the techno-functional characteristics of purslane proteins**

| **Property** | **WAC** | **OAC** | **FA** | **FS** | **EA** |
| --- | --- | --- | --- | --- | --- |
| **OAC** | -0.845** | 1 |  |  |  |
| **FA** | -0.963** | 0.904** | 1 |  |  |
| **FS** | -0.972** | 0.891** | 0.998** | 1 |  |
| **EA** | -0.929** | 0.970** | 0.975** | 0.971** | 1 |
| **ES** | -0.970** | 0.901** | 0.998** | 0.999** | 0.975** |

**Correlation is significant at the 0.01 and 0.05 levels

**Table S2** FTIR peaks assignment for defatted purslane flour (DF), protein concentrate (PC), and protein isolate (PI)

| **Functional group** | **DF** | **PC** | **PI** | **Suggested nutrients** |
| --- | --- | --- | --- | --- |
| C=O stretching | 1640 | 1641 | 1636 | Protein amide-Ι band |
| N–H bending; C–H stretching | 1528 | 1533 | 1528 | Protein amide-ΙΙ band |
| N–H stretching | 1386 | 1390 | 1389 | Protein amide-ΙΙΙ band |
| P=O stretching asymmetric; C-N stretching amine | 1247 | 1242 | 1242 | Nucleic acids |
| Intermolecular bonded O-H stretching; N-C stretching | 3322 | 3286 | 3291 | Protein and poly-hydroxyl compounds |
